# Dose-Dependent Shift in Relative Contribution of Homologous Recombination to DNA Repair after Low-LET Ionizing Radiation Exposure: Empirical Evidence and Numerical Simulation

**DOI:** 10.3390/cimb45090465

**Published:** 2023-09-09

**Authors:** Oleg Belov, Anna Chigasova, Margarita Pustovalova, Andrey Osipov, Petr Eremin, Natalia Vorobyeva, Andreyan N. Osipov

**Affiliations:** 1 Joint Institute for Nuclear Research, 6 Joliot-Curie St., 141980 Dubna, Russia; oleg.belov@jinr.int; 2 Institute of Biomedical Problems, Russian Academy of Sciences, 76A Khoroshevskoye Shosse, 123007 Moscow, Russia; 3 Institute of System Analysis and Management, Dubna State University, 19 Universitetskaya St., 141980 Dubna, Russia; 4 N.N. Semenov Federal Research Center for Chemical Physics, Russian Academy of Sciences, 119991 Moscow, Russia; annagrekhova1@gmail.com (A.C.); a-2-osipov@yandex.ru (A.O.); nuv.rad@mail.ru (N.V.); 5 Emanuel Institute for Biochemical Physics, Russian Academy of Sciences, 119334 Moscow, Russia; 6 State Research Center—Burnasyan Federal Medical Biophysical Center of Federal Medical Biological Agency (SRC—FMBC), 123098 Moscow, Russia; pustovalova.mv@mipt.ru; 7 School of Biological and Medical Physics, Moscow Institute of Physics and Technology, 141700 Dolgoprudny, Russia; 8 FSBI “National Medical Research Center for Rehabilitation and Balneology”, Ministry of Health of Russia, 121099 Moscow, Russia; ereminps@gmail.com

**Keywords:** DNA double-strand breaks, ionizing radiation, DNA repair pathways, homologous recombination, mathematical modeling

## Abstract

Understanding the relative contributions of different repair pathways to radiation-induced DNA damage responses remains a challenging issue in terms of studying the radiation injury endpoints. The comparative manifestation of homologous recombination (HR) after irradiation with different doses greatly determines the overall effectiveness of recovery in a dividing cell after irradiation, since HR is an error-free mechanism intended to perform the repair of DNA double-strand breaks (DSB) during S/G2 phases of the cell cycle. In this article, we present experimentally observed evidence of dose-dependent shifts in the relative contributions of HR in human fibroblasts after X-ray exposure at doses in the range 20–1000 mGy, which is also supported by quantitative modeling of DNA DSB repair. Our findings indicate that the increase in the radiation dose leads to a dose-dependent decrease in the relative contribution of HR in the entire repair process.

## 1. Introduction

Ionizing radiation (IR) affects the DNA structure by inducing various types of damage, of which DNA double-strand breaks (DSB) are considered to be the most deleterious examples [1,2]. DSBs are considered to be critical DNA lesions because their misrepair can lead to severe mutations, oncogenesis, cell death or senescence [3]. In this regard, the selection of an optimal repair pathway is crucial to the cell in terms of achieving a final outcome [4]. Non-homologous end joining (NHEJ) and homologous recombination (HR) are two major DSB repair pathways present in mammalian and human cells. NHEJ fuses the two broken ends with little regard for homology, leading to deletions and other rearrangements [5]. In contrast, HR typically copies the missing information from the sister chromatid into the break site, resulting in the exact reconstitution of the original sequence. In the last few decades, at least three alternative pathways of DSB repair were suggested to be operational following the ionizing radiation exposure. These distinct pathways, namely single-strand annealing (SSA), microhomology-mediated end-joining (MMEJ) and alternative end-joining (Alt-EJ), are distinguished based on the amount of DNA sequence complementarity used to align DNA ends [6].

Revealing the mechanistic nature of DSB repair pathways and evaluating their manifestation in response to different radiation doses aims to improve our understanding of the deleterious effects of ionizing radiation and our ability to predict them [7,8]. The development of our predictive capabilities impacts the accuracy of radiotherapy and radiation-induced cancer risk estimations, as well as being relevant to health issues in people living in areas with high background radiation and future manned space exploration, as astronauts can be exposed to complex radiation fields.

One of the initial events involved in the complex process of DNA repair from DSBs is the phosphorylation of the core histone H2AX by kinases of the PIKK families (phosphatidylinositol 3 kinase-related kinases) ATM, ATR and DNA-PKcs in the flanking regions of DSBs of chromatin [9]. Dynamic microstructures containing thousands of copies of the phosphorylated histone H2AX (γ-H2AX), which are called “foci” in the literature, are easily visualized using immunocytochemical staining [10,11,12]. The analysis of γH2AX foci provides information regarding the number of DNA repair sites derived from DSBs, their distribution over the nuclear volume, and their impact on the kinetics of repair. However, it is equally important to evaluate not only changes in the total number of DSBs, but also the proportion of DSBs repaired via the HR error-free pathway. For this purpose, the analysis of the foci associated with the key HR protein Rad51 is most often used [13].

Computational and mathematical modeling associated with cancer radiotherapy and radiation risk assessments have been undertaken for decades, and they proceed along with the new biophysics phenomena that have been identified [14,15,16,17,18]. Along with the modeling of radiation risk itself, there are a series of models designed to understand the kinetics of radiation-induced DNA damage repair and associated secondary cancer risk. Combining the numerical simulation with experimental methods allows us to identify new mechanistic properties involved in the DNA repair process, which are hardly accessible to experimental studies, but could be important in terms of providing a detailed understanding of the variety of outcomes induced by ionizing radiation.

One of the intriguing tasks potentially being solved by combining the experimental measurements with simulation techniques is the activation of specific DSB pathways following low-dose radiation exposure. Currently, there are many works devoted to changes in the number of DNA damage foci present in mammalian cells irradiated at low doses [19,20,21,22]. However, the issue of DSB repair efficiency after low-dose radiation exposure remains one of the controversial topics of present-day radiation biology.

The current study aims to perform the experimental probing of the relative contribution of the HR pathway to DSB repair, which is induced via X-ray exposure in the dose range from 20 to 1000 mGy, with the subsequent numerical evaluation of γ-H2AX foci considered to be the main DSB repair marker.

## 2. Materials and Methods

### 2.1. Experimental Study

#### 2.1.1. Cell Culture

The studies were performed using a human skin fibroblast culture (Cell Applications, San Diego, CA, USA, Cat.no. 106K-05a). Cells were cultured at 37 °C and 5% CO_2_ in a standard DMEM culture medium with a high glucose content (4.5 g/L) (Thermo Fisher Scientific, Waltham, MA, USA) supplemented with 2 mmol/L of L-glutamine (Thermo Fisher Scientific, Waltham, MA, USA), 100 U/mL of penicillin, 100 μg/mL of streptomycin (Thermo Fisher Scientific, Waltham, MA, USA) and 10% fetal bovine serum (Thermo Fisher Scientific, Waltham, MA, USA). Fibroblasts were then plated on 4-centimeter-squared coverslips in 35-millimeter Petri dishes (SPL Lifesciences, Pocheon-City, Gyeonggi-Do, Republic of Korea) with a density of 10^4^ cells/cm^2^. Next, 3–5 passages of cells in the phase of exponential growth (cell population density ~60–70%) were used to perform the experiments.

#### 2.1.2. Irradiation

The cells were irradiated using the RUB RUST-M1 X-irradiator (Diagnostika-M LLC, Moscow, Russia), which was equipped with two X-ray emitters, at a dose rate of 40 mGy/min and voltage of 100 kV, current of 0.8 mA, using a 1.5-millimeter Al filter and at a temperature of 4 °C (LAB ARMOR BEADS thermal granules were used for cooling). The error in the exposure dose was estimated to be within 15%. After irradiation, cells were incubated under the standard conditions of a CO_2_ incubator (37 °C, 5% CO_2_) for 0.25–24 h.

#### 2.1.3. Immunocytochemistry

Cells were fixed on coverslips in 4% paraformaldehyde in PBS (pH 7.4) for 15 min at room temperature, followed by two PBS rinses (pH 7.4) and permeabilization in 0.3% Triton-X100 (in PBS, pH 7.4) supplemented with 2% bovine serum albumin (BSA) to block non-specific antibody binding. To perform immunocytochemical staining, the slides were incubated for 1 h at room temperature with a primary mouse monoclonal antibody against γH2AX (dilution 1: 200, 05-636-I clone JBW301, Merck-Millipore, Burlington, VT, USA) and rabbit polyclonal antibody against Rad51 (dilution 1:200, ABE257, Merck-Millipore, Burlington, VT, USA) or CENPF (dilution dilution 1:200, ab5, Abcam, Waltham, MA, USA) in PBS (pH 7.4) containing 1% BSA. After rinses with PBS, cells were incubated for 1 h at room temperature with the goat anti-mouse Alexa Fluor 488 conjugated secondary antibodies IgG (H + L) (Life Technologies, Carlsbad, SA, USA), with a dilution of 1: 600 and goat anti-rabbit rhodamine conjugated antibodies (Merck-Millipore, Burlington, VT, USA), with a dilution of 1:400 in PBS (pH 7.4) with 1% BSA. ProLong Gold medium (Life Technologies, Carlsbad, SA, USA) was used with DAPI to perform DNA counter-staining and for the prevention of photo fading. Cells were viewed and imaged using a Nikon Eclipse Ni-U microscope (Nikon, Tokyo, Japan) equipped with a high definition ProgRes MFcool camera (Jenoptik AG, Jena, Germany). The filter sets UV-2E/C (340–380-nanometer excitation and 435–485-nanometer emission), B-2E/C (465–495-nanometer excitation and 515–555-nanometer emission) and Y-2E/C (540–580-nanometer excitation and 600–660-nanometer emission) were used. At least 200 cells were analyzed to determine each data point. γ-H2AX and Rad51 foci were counted through manual scoring and using DARFI software (https://github.com/varnivey/darfi; accessed on 19 September 2016).

#### 2.1.4. Statistical Analysis

Statistical analysis of the data was conducted using the Statistica 8.0 software (StatSoft, Tulsa, OK, USA). The results were presented as the means of three independent experiments ± standard error (*SE*).

### 2.2. Evaluating the Percentage Contribution of HR to DSB Repair

**Figure 1:**
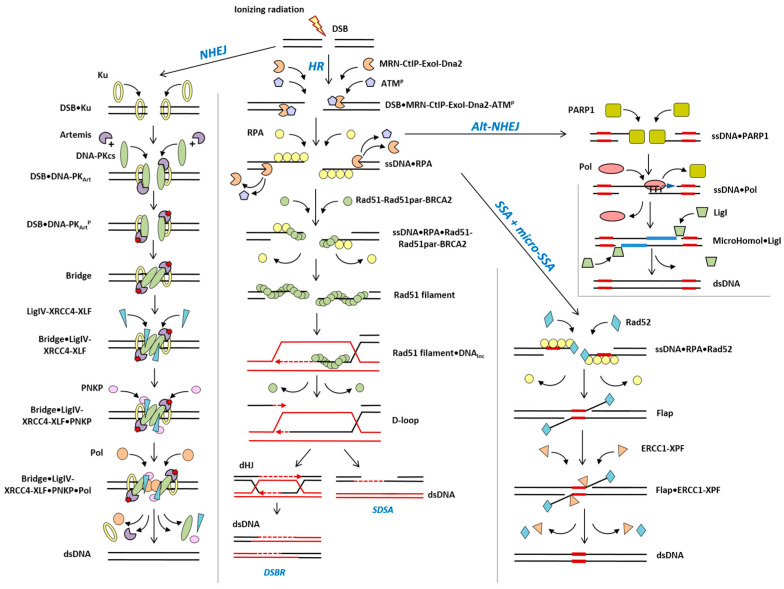
Pathways for DSB repair in mammalian and human cells.

In order to evaluate the percentage contribution of HR to the repair process, the quantitative model of radiation-induced DSB repair was used [23]. The model consisted of five main parts. The first part evaluated the initial yield of radiation-induced DSBs. The other parts were the quantitative models of NHEJ, HR, SSA and Alt-EJ repair pathways, as shown in Figure 1. To simulate the processing of DNA lesions by repair enzymes, the mass-action chemical kinetics approach was used.

The kinetics of DSB induction and repair were simulated as follows:

(1) $$\frac{dN_{0}}{dt}=\alpha (L)\frac{dD}{dt}N_{ir}-V_{\mathrm{NHEJ}}-V_{\mathrm{HR}}-V_{\mathrm{SSA}}-V_{\mathrm{micro}-\mathrm{SSA}}-V_{\mathrm{Alt}-\mathrm{EJ}}$$

where *N*_0_ = *N*_ncDSB_ + *N*_cDSB_; *V*_NHEJ_, *V*_HR_, *V*_SSA_, *V*_micro-SSA_, and *V*_Alt-EJ_ are the terms characterizing the elimination of DSBs by the NHEJ, HR, SSA, micro-SSA, and Alt-EJ repair pathways, respectively. The details of these terms are given in Equation (A4) of Appendix B. In Equation (1), *N_ir_* is the fraction of irreparable DSBs, which corresponds to the level of γ-H2AX foci remaining in the cell 24 h post-irradiation. The rate of initial DSB induction was calculated as $dN_{0}/dt=\alpha (L)dD/dt$, using the same method that is used in [24,25,26]. Here, *D* is the dose of ionizing radiation (Gy), and *α*(*L*) is the slope coefficient of linear dose dependence, which describes the DSB induction per unit of dose (Gy^−1^ per cell).

Enzymatic interactions that occurred in the course of repair were simulated as follows: within the NHEJ pathway, the stage of Ku binding to a DSB was represented by the kinetic equation below

(2) $$\left[\mathrm{DSB}\right]\;+\;[\mathrm{Ku}]\overset{K_{1}}{\underset{K_{-1}}{⇄}}[\mathrm{DSB}•\mathrm{Ku}],$$

where quantities in brackets denote time-dependent intracellular concentrations of repair complexes, and *K* values with an appropriate subscript are used to represent the dimensional reaction rate-constants. Here, [DSB] is the number of DSBs that undergo binding by Ku, and [DSB•Ku] is the level of the resulting intermediate complex.

The stage of the recruitment of the DNA-dependent protein kinase catalytic subunit (DNA-PKcs) and Artemis to a DSB site was described as

(3) $$[\mathrm{DSB}•\mathrm{Ku}]\;+\;[\mathrm{DNA}\text{-}\mathrm{PKcs}]\overset{K_{2}}{\underset{K_{-2}}{⇄}}[\mathrm{DSB}•{\mathrm{DNA}\text{-}\mathrm{PK}}_{\mathrm{Art}}],$$

In this kinetic equation, [DNA-PK] denotes a complex of Ku and DNA-PKcs. Art indicates that the mentioned intermediate complex is formed in the presence of Artemis.

The autophosphorylation of DNA-PKcs was represented by

(4) $$[\mathrm{DSB}•{\mathrm{DNA}\text{-}\mathrm{PK}}_{\mathrm{Art}}]\overset{K_{3}}{\to }[\mathrm{DSB}•{\mathrm{DNA}\text{-}\mathrm{PK}}_{\mathrm{Art}}^{P}],$$

where the superscript P defines the phosphorylated DNA-PK product.

The subsequent end-bridging process was described as a junction of two ${[\mathrm{DSB}}_{\mathrm{NHEJ}}•{\mathrm{DNA}\text{-}\mathrm{PK}}_{\mathrm{Art}}^{P}]$ constructs formed at the previous repair stage.

(5) $$[\mathrm{DSB}•{\mathrm{DNA}\text{-}\mathrm{PK}}_{\mathrm{Art}}^{P}]\;+\;[\mathrm{DSB}•{\mathrm{DNA}\text{-}\mathrm{PK}}_{\mathrm{Art}}^{P}]\;\overset{K_{4}}{\underset{K_{-4}}{⇄}}\;\left[\mathrm{Bridge}\right],$$

Here, the [Bridge] intermediate complex characterizes the final product of the reaction.

The further assembly of the NHEJ repair complex promotes the recruitment of LigIV with its associated factors XRCC4 and XLF, as well as the subsequent involvement of the polynucleotide kinase phosphatase (PNKP) with a break site.

(6) $$\left[\mathrm{Bridge}\right]\;+\;[\mathrm{LigIV}\text{-}\mathrm{XRCC}4\text{-}\mathrm{XLF}]\;\overset{K_{5}}{\underset{K_{-5}}{⇄}}\;[\mathrm{Bridge}•\mathrm{LigIV}\text{-}\mathrm{XRCC}4\text{-}\mathrm{XLF}],$$

(7) $$[\mathrm{Bridge}•\mathrm{LigIV}\text{-}\mathrm{XRCC}4\text{-}\mathrm{XLF}]\;+\;[\mathrm{PNKP}]\;\overset{K_{6}}{\underset{K_{-6}}{⇄}}\;[\mathrm{Bridge}•\mathrm{LigIV}\text{-}\mathrm{XRCC}4\text{-}\mathrm{XLF}•\mathrm{PNKP}].$$

The final step of the NHEJ pathway implying the recruitment of a polymerase was denoted as follows:

(8) $$\begin{array}{l} {}[\mathrm{Bridge}•\mathrm{LigIV}\text{-}\mathrm{XRCC}4\text{-}\mathrm{XLF}•\mathrm{PNKP}]+[\mathrm{Pol}]\overset{K_{7}}{\underset{K_{-7}}{⇄}}[\mathrm{Bridge}•\mathrm{LigIV}\text{-}\mathrm{XRCC}4\text{-}\mathrm{XLF}•\mathrm{PNKP}•\mathrm{Pol}]\overset{K_{8}}{\to }\\ \;\;\;\;\;\;\;\;\;\;\;\overset{K_{8}}{\to }[\mathrm{dsDNA}]+[\mathrm{LigIV}\text{-}\mathrm{XRCC}4\text{-}\mathrm{XLF}]+[\mathrm{Pol}]+[\mathrm{PNKP}]+\;[\mathrm{DNA}\text{-}\mathrm{PKcs}]+[\mathrm{Ku}].\; \end{array}$$

In this consideration, after gap filling and ligation are finalized, it was accepted that the repair complexes dissociated, leaving the recovered double-stranded DNA (dsDNA).

The first stages of HR associated with the action of MRN, its co-factors (CtlP, ExoI, Dna2) and ATM were described in the model as follows:

(9) $$\left[\mathrm{DSB}\right]\;+\;[\mathrm{MRN}\text{-}\mathrm{CtIP}\text{-}\mathrm{ExoI}\text{-}\mathrm{Dna}2]\overset{P_{1}}{\underset{P_{-1}}{⇄}}[\mathrm{DSB}•\mathrm{MRN}\text{-}\mathrm{CtIP}\text{-}\mathrm{ExoI}\text{-}\mathrm{Dna}2],$$

(10) $$[\mathrm{ATM}]\overset{P_{2}}{\to }{[\mathrm{ATM}}^{P}],$$

(11) $$\begin{array}{l} {}[\mathrm{DSB}•\mathrm{MRN}\text{-}\mathrm{CtIP}\text{-}\mathrm{ExoI}\text{-}\mathrm{Dna}2]+[{\mathrm{ATM}}^{P}]\overset{P_{3}}{\underset{P_{-3}}{⇄}}[\mathrm{DSB}•\mathrm{MRN}\text{-}\mathrm{CtIP}\text{-}\mathrm{ExoI}\text{-}\mathrm{Dna}2•{\mathrm{ATM}}^{P}]\overset{P_{4}}{\to }\\ \;\;\;\;\;\;\;\;\;\;\;\;\;\;\;\;\;\;\;\;\;\;\;\;\;\;\overset{P_{4}}{\to }[\mathrm{ssDNA}]+[\mathrm{MRN}\text{-}\mathrm{CtIP}\text{-}\mathrm{ExoI}\text{-}\mathrm{Dna}2]+[{\mathrm{ATM}}^{P}], \end{array}$$

where the MRN (Mre11-Rad50-Nbs1) complex interacting with other repair factors is considered to be a single complex, and superscript P defines the phosphorylated species.

The involvement of the replication protein A (RPA) in eliminating the secondary structure of DNA and protecting single-stranded regions from other enzymatic activities was denoted by

(12) $$\left[\mathrm{ssDNA}\right]\;+\;[\mathrm{RPA}]\overset{P_{5}}{\underset{P_{-5}}{⇄}}[\mathrm{ssDNA}•\mathrm{RPA}].$$

The next HR step associated with formation of Rad51 filament was described via the following kinetic equation:

(13) $$\begin{array}{l} {}[\mathrm{ssDNA}\;•\;\mathrm{RPA}]\;+\;[\mathrm{Rad}51\text{-}\mathrm{Rad}51\mathrm{par}\text{-}\mathrm{BRCA}2]\overset{P_{6}}{\underset{P_{-6}}{⇄}}[\mathrm{ssDNA}\;•\;\mathrm{RPA}\;•\;\mathrm{Rad}51\text{-}\mathrm{Rad}51\mathrm{par}\text{-}\mathrm{BRCA}2]\overset{P_{7}}{\to }\\ \;\;\;\;\;\;\;\;\;\;\;\;\;\;\;\;\;\;\;\;\;\;\;\;\;\;\;\;\;\;\;\;\;\;\;\;\;\;\;\;\;\;\;\;\;\;\;\;\overset{P_{7}}{\to }\left[\mathrm{Rad}51\;\mathrm{filament}\right]\;+\;[\mathrm{RPA}],\;\; \end{array}$$

where the Rad51par abbreviation denotes five biologically important Rad51 paralogs (Rad51B, Rad51C, Rad51D, XRCC2 and XRCC3), and [Rad51 filament] defines the [ssDNA•Rad51-Rad51par-BRCA2] complex.

The formation of a displacement loop (D-loop) and two methods of its subsequent resolution were represented as follows:

(14) $$\begin{array}{l} {}[\mathrm{Rad}51\;\mathrm{filament}]\;+\;[{\mathrm{DNA}}_{\mathrm{inc}}]\overset{P_{8}}{\underset{P_{-8}}{⇄}}[\mathrm{Rad}51\;\mathrm{filament}\;•\;{\mathrm{DNA}}_{\mathrm{inc}}]\overset{P_{9}}{\to }\\ \;\;\;\;\;\;\;\;\;\;\;\;\overset{P_{9}}{\to }\left[D\text{-}\mathrm{loop}\right]\;+\;[\mathrm{Rad}51\text{-}\mathrm{Rad}51\mathrm{par}\text{-}\mathrm{BRCA}2], \end{array}$$

(15) $$\left[D\text{-}\mathrm{loop}\right]\;\overset{P_{10}}{\to }\left[\mathrm{dHJ}\right]\overset{P_{11}}{\to }\;\left[\mathrm{dsDNA}\right]\;+\;{[\mathrm{DNA}}_{\mathrm{inc}}],$$

(16) $$\left[D\text{-}\mathrm{loop}\right]\;\overset{P_{12}}{\to }\;\left[\mathrm{dsDNA}\right]\;+\;{[\mathrm{DNA}}_{\mathrm{inc}}],$$

where [DNA_inc_] is the incoming DNA duplex, $[\mathrm{Rad}51\;\mathrm{filament}•{\mathrm{DNA}}_{\mathrm{inc}}]$ complex is assumed to contain Rad54 protein, and dHJ is the double Holiday junction.

The SSA pathway was given as the below set of kinetics equations. The first step assuming interaction with Rad52 was denoted by

(17) $$[\mathrm{ssDNA}•\mathrm{RPA}]\;+\;[\mathrm{Rad}52]\overset{Q_{1}}{\underset{Q_{-1}}{⇄}}[\mathrm{ssDNA}•\mathrm{RPA}•\mathrm{Rad}52],$$

where the [ssDNA•RPA]  complex is the same as that shown in Equation (12).

The junction between Rad52 heptamer rings and each ssDNA termini that allowed the formation of a flapped structure is represented as follows:

(18) $$[\mathrm{ssDNA}•\mathrm{RPA}•\mathrm{Rad}52]\;+\;[\mathrm{ssDNA}•\mathrm{RPA}•\mathrm{Rad}52]\;\overset{Q_{2}}{\to }\;[\mathrm{Flap}]\;+\;[\mathrm{RPA}].$$

The cutting of the flapped ends by the ERCC1-XPF endonuclease and final ligation of a damaged site with LigIII complex were simulated via

(19) $$\;[\mathrm{Flap}]+[\mathrm{ERCC}1\text{-}\mathrm{XPF}]\overset{Q_{3}}{\underset{Q_{-3}}{⇄}}[\mathrm{Flap}•\mathrm{ERCC}1\text{-}\mathrm{XPF}]\overset{Q_{4}}{\to }[{\mathrm{dsDNA}}_{\mathrm{nicks}}]+[\mathrm{Rad}52]+[\mathrm{ERCC}1\text{-}\mathrm{XPF}],$$

(20) $$[{\mathrm{dsDNA}}_{\mathrm{nicks}}]\;+\;[\mathrm{LigIII}]\;\overset{Q_{5}}{\underset{Q_{-5}}{⇄}}\;[{\mathrm{dsDNA}}_{\mathrm{nicks}}•\;\mathrm{LigIII}]\;\overset{Q_{6}}{\to }\;[\mathrm{dsDNA}]\;+\;[\mathrm{LigIII}].$$

Here, Rad52 and ERCC1-XPF are assumed to dissociate from the processing site.

The simulation of the alternative end-joining pathways was based on the hypothesis suggesting two different Ku-independent repair mechanisms [27]. The first of these mechanisms was represented by MMEJ, which was sometimes considered to be an independent end-joining mechanism distinct from the other pathways. Meanwhile, experimental evidence suggested that this type of repair represented a specific subclass of the SSA pathway known as micro-SSA [28,29]. On the basis of these considerations, in our model, rejoining via MMEJ was simulated as the additional part of DSBs, which followed the SSA pathway.

To simulate the Alt-EJ pathway, an additional mechanistic explanation was proposed. According to the recent hypothesis, Alt-EJ required activity of MRX complex and possibly exhibited the same initiation steps as HR [27]. Therefore, the initial stages of Alt-EJ was described by Equations (9)–(11). After the production of ssDNA, the activity of PARP1 recruited to the DSB site was characterized by Equation (21).

(21) $$\left[\mathrm{ssDNA}\right]\;+\;[\mathrm{PARP}1]\;\overset{R_{1}}{\underset{R_{-1}}{⇄}}\;[\mathrm{ssDNA}•\mathrm{PARP}1]\;.$$

The kinetics of microhomology production via polymerase activity was simulated via

(22) $$[\mathrm{ssDNA}•\mathrm{PARP}1]\;+\;[\mathrm{Pol}]\;\overset{R_{2}}{\to }\;[\mathrm{ssDNA}•\mathrm{Pol}]\;+[\mathrm{PARP}1],$$

(23) $$[\mathrm{ssDNA}•\mathrm{Pol}]\overset{R_{3}}{\to }\;[\mathrm{MicroHomol}]\;+[\mathrm{Pol}].$$

In Equation (23), [MicroHomol] denotes the yield of microhomology.

The final step of Alt-EJ was believed to rely on LigI activity [27]. In the model, this stage was represented as follows:

(24) $$[\mathrm{MicroHomol}]+[\mathrm{LigI}]\overset{R_{4}}{\underset{R_{-4}}{⇄}}[\mathrm{MicroHomol}•\mathrm{LigI}]\overset{R_{5}}{\to }[\mathrm{dsDNA}]+[\mathrm{LigI}].$$

The kinetics of the induction of γ-H2AX foci was simulated by summing up all active forms of DNA-PKcs and ATM, which were considered in the model, which was similar to the method used in [24]

(25) $$V_{\gamma \text{-}H2\mathrm{AX}+}=\frac{K_{9}\;[\mathrm{Sum}]\;[H2\mathrm{AX}]}{K_{10}+[\mathrm{Sum}]},$$

where [H2AX] is the level of the histone variant H2AX and

(26) $$\begin{array}{l} \;\;\;\;\;\;[\mathrm{Sum}]=[\mathrm{DSB}\;•\;{\mathrm{DNA}\text{-}\mathrm{PK}}_{\mathrm{Art}}^{P}]+[\mathrm{Bridge}]+[\mathrm{Bridge}\;•\;\mathrm{LigIV}\text{-}\mathrm{XRCC}4\text{-}\mathrm{XLF}]+\\ +\;[\mathrm{Bridge}\;•\;\mathrm{LigIV}\text{-}\mathrm{XRCC}4\text{-}\mathrm{XLF}\;•\;\mathrm{PNKP}]+[\mathrm{Bridge}•\mathrm{LigIV}\text{-}\mathrm{XRCC}4\text{-}\mathrm{XLF}•\mathrm{PNKP}•\mathrm{Pol}]+\\ \;\;\;\;\;\;\;\;\;\;\;\;\;\;\;\;\;\;\;\;\;\;\;\;\;\;\;+\;[\mathrm{DSB}•\mathrm{MRN}\text{-}\mathrm{CtIP}\text{-}\mathrm{ExoI}\text{-}\mathrm{Dna}2•{\mathrm{ATM}}^{P}]. \end{array}$$

The mechanism of γ-H2AX foci dephosphorylation was assumed to be proportional to the amount of repaired DNA, as well as its spontaneous decay with the corresponding rate constants *K*_11_ and *K*_12_.

(27) $$V_{\gamma \text{-}H2\mathrm{AX}-}=\;K_{11}[\mathrm{dsDNA}]+K_{12}[\gamma \text{-}H2\mathrm{AX}].$$

To describe the interactions between repair enzymes and their substrates, the mass-action chemical kinetics approach was used. The details of the application of this approach to simulate the dynamic change in the intracellular concentrations of main repair complexes, as well as the model equations and their parameters, are described in Appendix A, Appendix B, Appendix C.

The evaluation of the percentage contribution of HR to DSB repair was performed via the calculated time-courses of γ-H2AX and Rad51 foci for the particular dose of X-rays. The HR contribution to PHR was evaluated as the following ratio:

(28) $$P_{\mathrm{HR}}=100\times \bar{y_{9}}/\;\bar{x_{14}},$$

where $\bar{y_{9}}$ and $\bar{x_{14}}$ are the mean numbers of Rad51 and γ-H2AX foci, respectively, counted via a 24-h simulation period.

To obtain a dependence of PHR on the X-ray dose, the ODE system was run using a sufficiently small dose step equal to 0.1 mGy within the range 0–1000 mGy. This simulation procedure yielded a curve of PHR (*D*) dependence that was expressed by the following formula:

(29) $$P_{\mathrm{HR}}(D)=100\times \bar{y_{9}(D)}/\;\bar{x_{14}(D)}.$$

## 3. Results

### 3.1. Experimental Results

**Figure 2:**
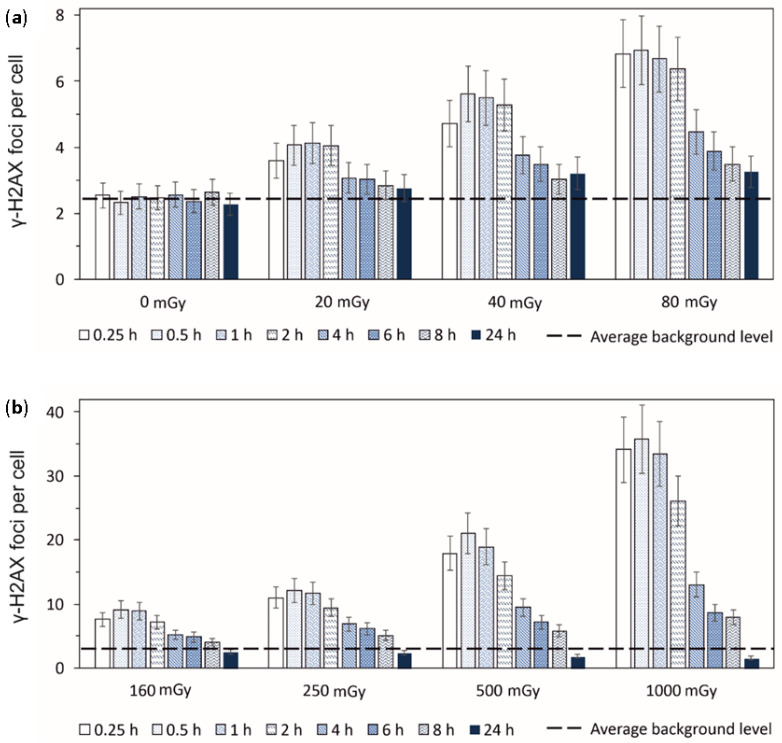
Experimentally measured time-courses of γ-H2AX foci remaining in normal human skin fibroblasts after exposure to (**a**) 20 mGy, 40 mGy, or 80 mGy and (**b**) 160 mGy, 250 mGy, 500 mGy or 1000 mGy of X-rays in comparison to background levels used as control (±*SE*).

In Figure 2, γ-H2AX foci yields are shown, as scored at 0.25–24 h after exposure to different doses of X-rays ranging from 20 to 1000 mGy. Foci yields scored in cells exposed to 500 mGy and 1000 mGy tend to peak at 0.25–1.0 h, while exposure to doses of 20–250 mGy resulted in maximum levels of foci being found at 0.25–2.0 h. At a point closer to 4 h after exposure, the signal of γ-H2AX sharply decreased with time due to the completion of the fast DSB repair being a feature of NHEJ. Then, up to 24 h after exposure, the slow DSB repair occurred, which was typically associated with complex DSBs being repaired via the HR pathway.

It is noteworthy that 24 h after exposure to 40–80 mGy, the level of γ-H2AX foci does not drop to the control values, indicating a slowdown in the DSB repair kinetics following low doses of ionizing radiation. On the other hand, 24 h after exposure to 500 mGy and 1000 mGy, the observed levels of γ-H2AX foci were slightly below those of the control values. A possible explanation is that the exposure to low and moderate doses of ionizing radiation affects cell proliferation in an opposite manner. Low doses stimulate cell proliferation, while relatively high doses lead to their reduction.

One of the sources of DNA DSBs used in the proliferation of cells is the collapse of replication forks in the S phase of the cell cycle [30]. The repair of DSBs induced in this process follows the HR pathway [30,31]. The histone H2AX in the S phase is mainly phosphorylated by the ATR kinase [32,33]. As a consequence, the percentage of cells in the S phase contributes to the average amount of γ-H2AX foci in asynchronous cell populations. This process leads to the overestimation of the γ-H2AX foci level in cells exposed to low doses and the underestimation of its presence in cells irradiated with moderate doses. The observed pattern generally meets the previous findings obtained using human fibroblasts and mesenchymal stromal cells exposed to X-rays [34,35].

**Figure 3:**
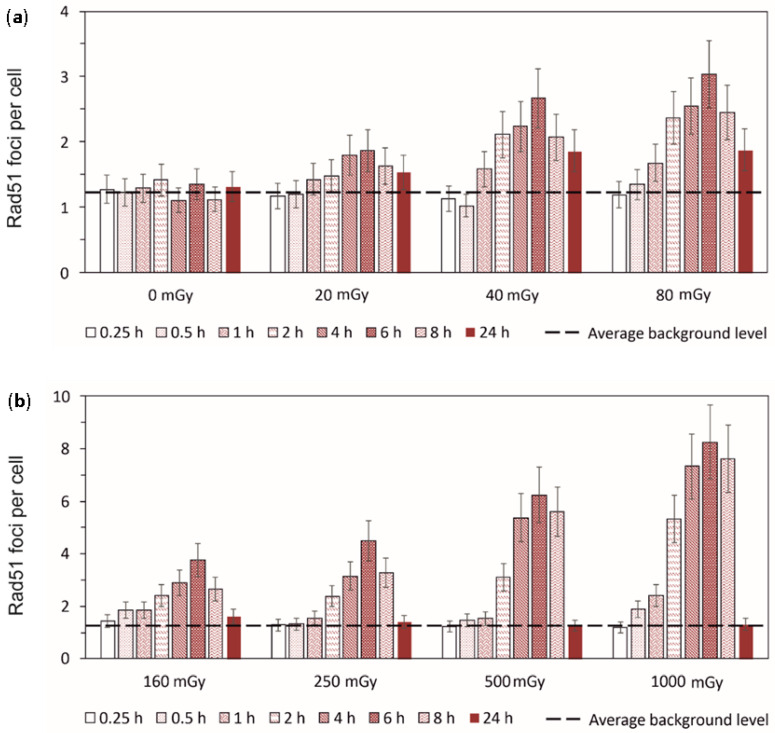
Experimentally measured time-courses of Rad51 foci remaining in normal human skin fibroblasts after exposure to (**a**) 20 mGy, 40 mGy or 80 mGy and (**b**) 160 mGy, 250 mGy 500 mGy or 1000 mGy of X-rays in comparison to background levels used as control (±*SE*).

To estimate the contribution of HR to the entire process of DNA DSB repair, we analyzed changes in the level of radiation-induced Rad51 foci, which are the key markers of HR (Figure 3). A statistically significant increase in the number of Rad51 foci was observed 2 h after irradiation, reaching its maximum values by 6 h. After 6 h, a decrease in the number of Rad51 foci was indicated. Finally, at 24 h after irradiation, the level of foci dropped down to the control values in cells exposed to 160–1000 mGy and remained higher than the control values in cells irradiated with doses of 40 mGy and 80 mGy.

**Figure 4:**
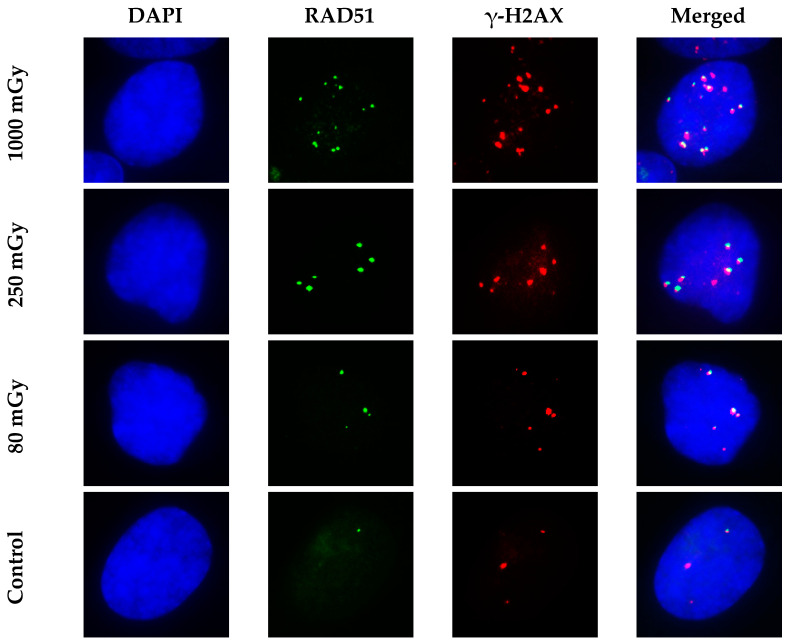
Representative images of RAD51 and γ-H2AX foci and their co-localization at 6 h post-irradiation. Fibroblasts were irradiated, and RAD51 and γ-H2AX were immunofluorescently labeled as described in Materials and Methods section. Nuclei were counter-stained with DAPI, as shown in blue in first column of images. RAD51 and γ-H2AX foci are shown in green and red, respectively. Images in the last column were produced by merging all three channels, and they show the co-localization patterns of RAD51 and γ-H2AX.

Figure 4 illustrates RAD51 and γ-H2AX foci and the co-localization of RAD51 foci with γ-H2AX foci in normal human skin fibroblasts at 6 h (maximum values of RAD51 foci) after X-ray irradiation with doses of 80, 250, and 1000 mGy. Dose-dependent increases in the number of foci of both proteins, as well as a perfect co-localization of RAD51 foci with γ-H2AX foci, are clearly seen on the microscopic images shown in Figure 4.

### 3.2. Percent Contribution of HR to DSB Repair

**Figure 5:**
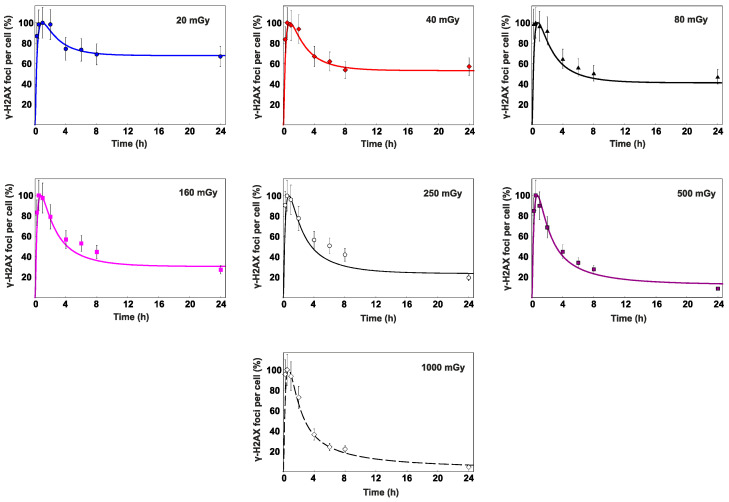
Time-courses of γ-H2AX foci remaining in normal human skin fibroblasts after exposure to different doses of X-rays. The curves are the calculated results; the symbols are the experimental data (±*SE*). The data are normalized based on the maximum number of foci observed within the 24-h period.

**Figure 6:**
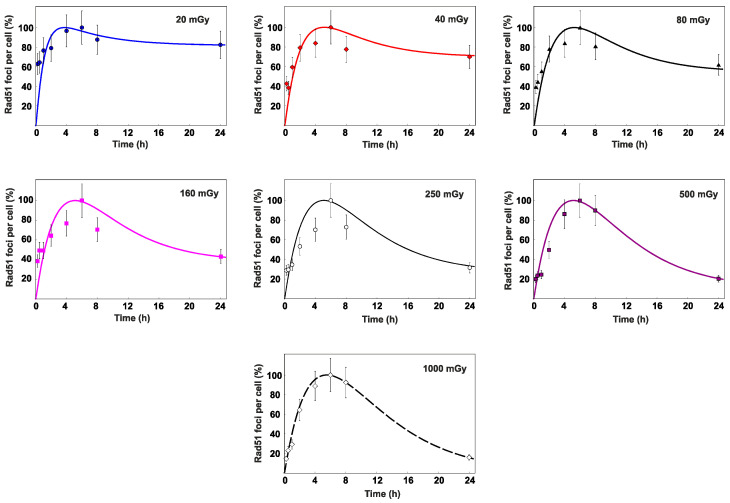
Time-courses of Rad-51 foci remaining in normal human skin fibroblasts after exposure to different doses of X-rays. The curves are the calculated results; the symbols are the experimental data (±*SE*). The data are normalized based on maximum number of foci observed within the 24-h period.

To confirm the DSB repair model validity of radiation doses in the range of 20–1000 mGy, the calculated time-courses of γ-H2AX and Rad51 foci were compared to the experimental data of normal human skin fibroblasts exposed to X-rays at doses of 20, 40, 80, 160, 250, 500 and 1000 mGy. The results of comparison are shown in Figure 5 and Figure 6, which depict kinetics of these foci in the range 0–24 h after irradiation.

Although the exposure to doses of 20–250 mGy results in relatively low absolute levels of γ-H2AX, the entire repair process does not seem to occur faster than in the case of higher considered doses. The proportion of γ-H2AX foci observed after exposure to 20–250 mGy remains elevated for a period similarly long to that following the exposure to the doses of 500–1000 mGy. We hypothesize that the observed pattern of DSB repair occurs due to the preferential activation of HR pathway, which takes longer to eliminate the damage.

Interestingly, following the exposure to doses of 160 mGy and above, the levels of γ-H2AX foci remaining 24 h after irradiation were lower than the background value. This finding suggests that DSBs normally induced as a consequence of non-radiation factors can undergo a more effective elimination if cells enter the process of radiation-induced DSB repair. It could also be expected that in cells that have successfully completed the repair of a certain portion of radiation-induced DSBs, the background levels of γ-H2AX foci can be suppressed compared to non-irradiated cells for at least a residual period after exposure.

Overall, the juxtaposition of the simulated and experimentally measured data affirms the validity of the suggested DSB repair model used for relatively low radiation doses, enabling the calculation of time-courses of γ-H2AX and Rad51 foci within the full dose range of interest (0–1000 mGy) to obtain a continuous dependence of *P_HR_* on *D*.

**Figure 7:**
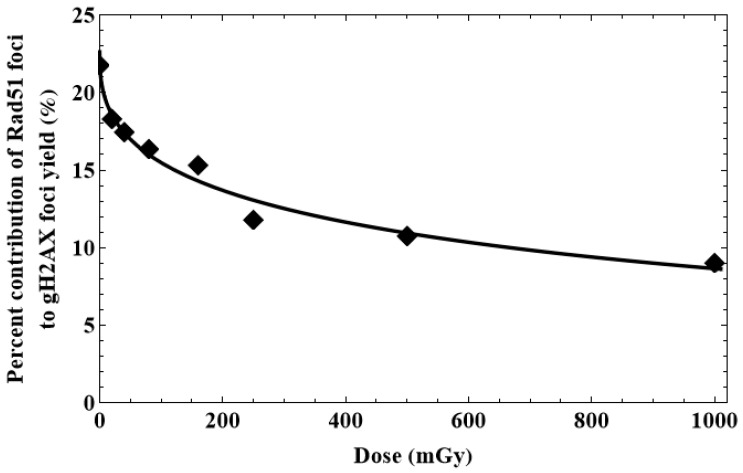
Dependence of the percentage contribution of HR to DSB repair on the radiation dose (*P_HR_*(*D*)). The curve line is fitted; the symbols are the experimental data.

The results yielding the percentage contribution of HR to DSB repair are shown in Figure 7. The general pattern of HR involvement in the DSB repair is characterized by a near-exponentially decreasing function, which is supposed to depend on the cell line, type of ionizing radiation and other factors. The dependence of HR on different factors requires more detailed examination.

### 3.3. Dose-Dependent Changes in the S/G2-Phase Cell Fractions

DNA repair via HR predominantly occurs in the S/G2 phases of the cell cycle. Therefore, to ensure the correct interpretation of the obtained results, it was important to estimate the changes in the proportion of cells in the S/G2 phases in the irradiated cell populations. For this purpose, we used immunohistochemical analysis of a protein marker of cells in S/G2 phases—Centromere protein F (CENPF). This protein, being a component of the nuclear matrix during G2 phase, has been used as a marker of S/G2 cells [36,37]. Its synthesis commences in the early S phase and ceases in the M phase, with a peak at the G2 phase [37].

**Figure 8:**
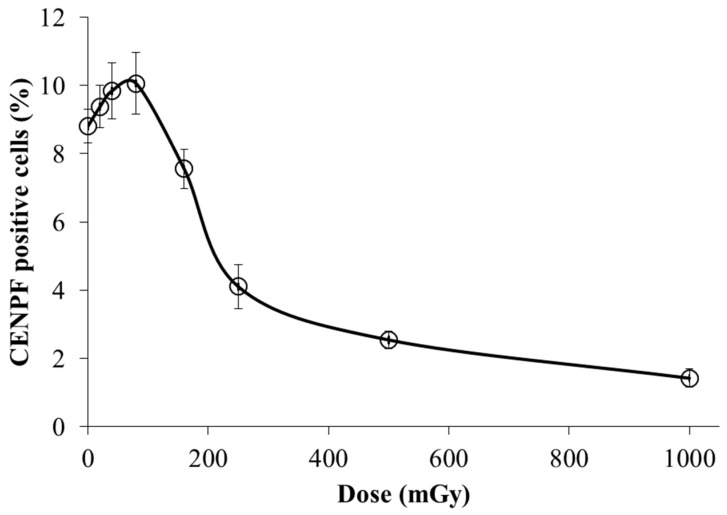
Dose-dependent changes in the S/G2-phase cell fractions (CENPF positive cells) 24 h after the irradiation of fibroblasts. Data are means ± SE of the three independent experiments.

The results presented in Figure 8 show that exposure to low doses (20–80 mGy) of X-rays leads to a slight increase in the proportion of CENPF-positive cells. However, these changes are not statistically significant (*p* > 0.05). In contrast, irradiation at doses of 250, 500 and 1000 mGy leads to significant 2.15 (*p* = 0.004), 3.69 (*p* = 0.0003) and 6.93 (*p* = 0.0002) fold decreases in the proportion of CENPF positive cells, respectively. In general, after irradiation at doses of 160–1000 mGy, the pattern of change in the proportions of S/G2-phase fibroblasts is characterized by a near-exponentially decreasing function. The obtained results are in good agreement with dose-dependent changes in the relative contribution of HR in irradiated cells.

## 4. Discussion

Our study reveals distinct patterns of γ-H2AX and Rad51 foci dynamics following the application of low and moderate doses of X-rays. We identified several parameters of the foci dynamics, which demonstrate different regularities in response to low and moderate doses, including periods of reaching peak levels of γ-H2AX foci, numbers of γ-H2AX and Rad51 foci remaining 24 h post-irradiation compared to control levels. All of these characteristics of DNA DSB repair demonstrate a pronounced dose-dependent shift. Within the dose steps selected for use in the analysis, the dose-dependent shift associated with reaching peak levels of γ-H2AX foci is observed between 250 and 500 mGy. The doses triggering the levels of remaining γ-H2AX and Rad51 foci appeared to be within the ranges of 80—500 mGy and of 80—160 mGy, respectively.

The juxtaposition of our results with other findings [38] suggests another confirmation of a dose-dependent shift in the activity of DNA repair systems, at least with regard to the repair of radiation-induced DSBs. On one hand, the number of DSBs increases linearly with the radiation dose, and the entire cell response depends on the correctness of DNA repair. Our results show that relatively low doses of low-LET ionizing radiation lead to a higher contribution of the error-free repair via the HR pathway. Since HR takes longer than NHEJ, low-dose-mediated activation of DNA repair mechanisms may not only protect DNA from the immediate damage, but also result in prolonged adaptive responses, protecting the cell from future oxidative insults (such as high-dose radiation), as is confirmed in [38,39,40,41,42,43,44]. The observed shift to error-free HR allows the restoration of the genome with maximum fidelity.

The mechanistic nature of the relative contribution of HR at low doses remains under debate. Nevertheless, our results support one of the possible scenarios of engagement of this repair pathway in the response to low doses of low-LET ionizing radiation [45]. According to these findings, error-prone pathways, including NHEJ, Alt-EJ and SSA, are partly or completely suppressed and likely only operate when HR fails to process the damage. An increase in the dose of low-LET radiation leads to the suppression of HR via mechanisms that remain to be identified, while the contribution of NHEJ increases and becomes the first choice. The decrease in the fraction of cells in the S/G2 phases after irradiation at doses of 250–1000 mGy, as shown in our work, suggests that the simplest mechanism used to reduce the contribution of HR with increasing radiation dose is the cell cycle arrest in G1/S phase. It is believed that HR is primarily active in the S/G2 phase of the cell cycle, as HR requires a sister chromatid to perform template-based repair [46,47,48]. Thus, a simple mechanistic decrease in the portion of S/G2 cells should also lead to a decrease in the relative HR contribution to DNA DSB repair. However, to prove this assumption, additional research is needed to address the role played by HR proteins in cell cycle regulation, including the analysis of this regulation in low-dose radiation-exposed cells. To determine if the HR contribution in DNA DSB repair is directly affected by radiation doses, the analysis should be restricted to S/G2 cells. The overall strategy for the future research in this direction should elucidate whether the proportion of the cells with the cell cycle arrest is really comparable to the proportion of cells in which the direct effect of radiation on the HR-capacity is seen. Our study was limited to a radiation dose of 1 Gy, while the model in [45] postulates that DNA end resection remains active, showing signs of suppression only above 20 Gy. Therefore, the increased engagement of error-prone Alt-EJ and SSA under conditions of persisting resection and partially suppressed HR can only be identified through the simulation approach.

Considering that there are plenty of well-established findings regarding the strong compensatory power of the DBS repair in eukaryotes, it is incorrect to assume that the radiation effects associated with the DSB removal follow linear dose dependence. Even the mechanistic nature of the assembly of DSB repair super-complexes in response to the appearance of radiation-induced lesions reflects the non-linear kinetics of this process, which may blur the resulting outcome within a certain dose range. It may also suggest that genotoxicity and late risks associated with the quality of DSB repair after exposure to low-LET radiation can cross some radiation dose threshold.

## Data Availability

Not applicable.

## Appendix A. Details the DSB Repair Model

The initial yield of DSBs, denoted as (*N*_0_), was calculated as

(A1) $$\frac{dN_{0}}{dt}=\alpha (L)\frac{dD}{dt},$$

where *D* is the radiation dose (Gy), and α(L)=aexp(−bL) is the slope coefficient of linear dose dependence, which describes DSB induction per unit of dose (Gy^−1^ per cell). Parameters *a* and *b* used in this equation are presented in Appendix C. The particular details of parameter evaluation, as well as the simulation of each repair pathway, can be found in [23].

The dynamic change in the intracellular concentrations of the main repair complexes is expressed via the following differential equation:

(A2) $$\frac{dX}{dt}=V_{+}(X_{i},N_{0})-V_{-}(X_{i},N_{0}),$$

where $X_{i}$ (i=1,…,n) is the intracellular level of the *i*-th repair complex, t is time, and the functions $V_{+}$ and $V_{-}$ are the complex accumulation and degradation, respectively. The dimensionless form of the system of ordinary differential equations (ODE) referred to the simulation of NHEJ, HR, SSA and two alternative repair pathways, as well as its parameters and initial conditions, which are presented in Appendix B, Appendix C. In the present study, the ODE system was solved using the fourth-order Runge–Kutta method. The integration time-step is denoted as 10^−10^ s in order to satisfy the fastest reactions being simulated in the model.

In order to simulate DSB rejoining in the asynchronous cell culture, we set a model cell cycle distribution similar to the distribution of HF19 cells observed in [49], where 40% of cells were in G_0_/G_1_, <10% were in S phase and 50% were in G_2_/M. Assuming that S-phase cells include two equal sub-fractions of early S and late S cells, the final distribution was set as follows: 45% of cells are in the G_0_/G_1_ and early S phases, and 55% of cells are in the late S and G_2_/M phases. Based on the data discussed in [26,28,50,51], the model provides the following pathways for the repair of non-clustered and clustered DSBs: In G_0_/G_1_ and early S phases, non-clustered DSBs are likely to follow rejoining via NHEJ, while the limited number of complex DSBs may undergo PARP1-dependent Alt-EJ. The other pathways are assumed to be unavailable or masked by NHEJ in these phases. In the late S and G_2_/M phases, non-clustered DSBs may follow NHEJ and be a substrate for SSA and micro-SSA pathways. The clustered DSBs are suggested to proceed through HR, SSA and Alt-EJ. Finally, in our calculations, the models of corresponding repair mechanisms are processed based on the following scheme:

(A3) $$G_{0}/G_{1},\;\mathrm{and}\;\mathrm{early}\;S\;\;\left\{\begin{array}{l} 0.45\;TN_{\mathrm{ncDSB}}\to \;\mathrm{NHEJ},\\ 0.45\;TN_{\mathrm{cDSB}}\to \mathrm{Alt}\text{-}\mathrm{EJ}, \end{array}\right.\;\mathrm{late}\;S\;\mathrm{and}\;G_{2}/M\;\left\{\begin{array}{l} 0.55\;TN_{\mathrm{ncDSB}}\to \;\mathrm{NHEJ},\;\mathrm{SSA},\\ 0.55\;TN_{\mathrm{cDSB}}\to \mathrm{HR},\;\mathrm{SSA},\;\mathrm{Alt}\text{-}\mathrm{EJ}, \end{array}\right.$$

where 0.45 and 0.55 represent the fractions of cells in the G_0_/G_1_ and early S, as well as in the late S and G_2_/M, phases, respectively; *N*_ncDSB_ and *N*_cDSB_ represent amounts of non-clustered and clustered DSBs; and *T* represents the total size of the exponentially growing cell culture set to be 10^5^ cells, as mentioned in [49]. The model accounts for the cell cycle dependence of the repair pathways by initiating the computation procedure with the levels of *N*_ncDSB_ and *N*_cDSB_ parameters based on the cell cycle distribution of the cell culture used in the experiment.

## Appendix B. Equations of the Model

The dynamic change in the levels of NHEJ repair complexes and γ-H2AX foci is described by the following system of ordinary differential equations:

(A4) $$\begin{array}{c} \frac{dn_{0}}{d\tau }=\alpha (L)\frac{dD}{dt}N_{ir}-n_{0}(k_{1}x_{1}+p_{1}y_{1})+k_{-1}x_{2}+p_{-1}y_{2},\\ \frac{dx_{2}}{d\tau }=k_{1}N_{0}x_{1}-x_{2}(k_{-1}+k_{2}x_{3})+k_{-2}x_{4},\\ \frac{dx_{4}}{d\tau }=k_{2}x_{2}x_{3}-x_{4}(k_{3}+k_{-2}),\\ \frac{dx_{5}}{d\tau }=k_{3}x_{4}-k_{4}x_{5}^{2}+k_{-4}x_{6},\\ \frac{dx_{8}}{d\tau }=k_{-6}x_{10}+k_{5}x_{6}x_{7}-x_{8}(k_{-5}+k_{6}x_{9}),\\ \frac{dx_{10}}{d\tau }=k_{-7}x_{12}+k_{6}x_{8}x_{9}-x_{10}(k_{-6}+k_{7}x_{11}),\\ \frac{dx_{12}}{d\tau }=k_{7}x_{10}x_{11}-x_{12}(k_{8}+k_{-7}),\\ \frac{dx_{13}}{d\tau }=k_{8}x_{12}+p_{12}y_{14}+p_{11}y_{15}+q_{6}z_{8}+r_{5}w_{7},\\ \frac{dx_{14}}{d\tau }=\frac{k_{9}(x_{5}+x_{6}+x_{8}+x_{10}+x_{12})\cdot x_{15}}{k_{10}+x_{5}+x_{6}+x_{8}+x_{10}+x_{12}}-k_{11}x_{13}-k_{12}x_{14}. \end{array}$$

The initial conditions of this system of wild-type cells are as follows: *n*_0_(0) = α(L)D, *x*_2_(0) = *x*_4_(0) = *x*_5_(0) = *x*_6_(0) = *x*_8_(0) = *x*_10_(0) = *x*_12_(0) = *x*_13_(0) = *x*_14_(0) = 0. The values of variables *x*_1_, *x*_3_, *x*_7_, *x*_9_, *x*_11_ and *x*_15_ are set to be constant and equal to *x*_1_.

In Equation (A4), *n*_0_ is the scaled number of radiation-induced DBSs; *N_ir_* is the non-dimensional fraction of irreparable DSBs; *x*_1_, *x*_3_, *x*_7_, *x*_9_ and *x*_11_ are scaled intracellular concentrations of the Ku, DNA-PKcs, LigIV-XRCC4-XLF, PNK and Pol enzymes, respectively; *x*_2_, *x*_4_, *x*_5_, *x*_6_, *x*_8_, *x*_10_, *x*_12_ and *x*_13_, are normalized intracellular concentrations of intermediate complexes represented by [DSB•Ku], $[\mathrm{DSB}•{\mathrm{DNA}\text{-}\mathrm{PK}}_{\mathrm{Art}}]$, $[\mathrm{DSB}•{\mathrm{DNA}\text{-}\mathrm{PK}}_{\mathrm{Art}}^{P}]$, [Bridge], [Bridge•LigIV-XRCC4-XLF], [Bridge•LigIV-XRCC4-XLF•PNKP], [Bridge•LigIV-XRCC4-XLF•PNKP•Pol] and [dsDNA]; *x*_14_, *x*_15_ and *y*_5_ are the scaled levels of the γ-H2AX foci, histone variant H2AX and $[\mathrm{DSB}•\mathrm{MRN}\text{-}\mathrm{CtIP}\text{-}\mathrm{ExoI}\text{-}\mathrm{Dna}2•{\mathrm{ATM}}^{P}]$ complex that contribute to the fluorescent signal upon the measurement of γ-H2AX foci induction; *y*_15_, *z*_8_ and *w*_5_ are the concentrations of the [dHJ], $\left[{\mathrm{dsDNA}}_{\mathrm{nicks}}•\mathrm{LigIII}\right]$ and [MicroHomol•LigI], respectively; and *k_i_* is the scaled rate constants. The variables of the model are normalized based on Ku total cellular level: $n_{0}=N_{0}/X_{1}$, $x_{i}=X_{i}/X_{1}$, $y_{i}=Y_{i}/X_{1},$ $z_{i}=Z_{i}/X_{1}$ and $w_{i}=W_{i}/X_{1}$. In terms of molar concentration, this level was estimated as $X_{1}=N/(N_{A}V_{nucl})=9.19\times 10^{-7}M$, where *N* = 400 000 is the number of Ku molecules per cell [52], *N_A_* is the Avogadro constant and $V_{nucl}=7.23\times 10^{-13}\;L$ is an average volume of the cell nucleus in human fibroblasts [53]. In this consideration, *x*_1_ = 1.

The kinetics of the HR repair complexes is described by the following system of ordinary differential equations:

(A5) $$\begin{array}{c} \frac{dy_{2}}{d\tau }=p_{1}n_{0}y_{1}-y_{2}(p_{-1}+p_{3}y_{4})+p_{-3}y_{5},\\ \frac{dy_{4}}{d\tau }=p_{2}y_{3}-y_{4}(p_{-2}+p_{3}y_{2})+y_{5}(p_{4}+p_{-3}),\\ \frac{dy_{5}}{d\tau }=p_{3}y_{2}y_{4}-y_{5}(p_{4}+p_{-3}),\\ \frac{dy_{6}}{d\tau }=p_{4}y_{5}-y_{6}(p_{5}y_{7}+r_{1}w_{1})+p_{-5}y_{8}+r_{-1}w_{2},\\ \frac{dy_{8}}{d\tau }=p_{-6}y_{10}+p_{5}y_{6}y_{7}-y_{8}(p_{-5}+p_{6}y_{9}+q_{1}z_{1})+q_{-1}z_{2},\\ \frac{dy_{10}}{d\tau }=p_{6}y_{8}y_{9}-y_{10}(p_{7}+p_{-6}),\\ \frac{dy_{11}}{d\tau }=p_{7}y_{10}-p_{8}y_{11}y_{12}+p_{-8}y_{13},\\ \frac{dy_{13}}{d\tau }=p_{8}y_{11}y_{12}-y_{13}(p_{9}+p_{-8}),\\ \frac{dy_{14}}{d\tau }=p_{9}y_{13}-y_{14}(p_{10}+p_{12}),\\ \frac{dy_{15}}{d\tau }=p_{10}y_{14}-p_{11}y_{15}. \end{array}$$

The initial conditions of this system for wild-type cells are as follows: *y*_2_(0) = *y*_4_(0) = *y*_5_(0) = *y*_6_(0) = *y*_8_(0) = *y*_10_(0) = *y*_11_(0) = *y*_13_(0) = *y*_14_(0) = *y*_15_(0) = 0. The values of *y*_1_, *y*_3_, *y*_7_, *y*_9_ and *y*_12_ variables are set to be constant and equal to *x*_1_.

In Equation (A5), *y*_1_, *y*_3_, *y*_4_, *y*_7_ and *y*_9_ are scaled intracellular concentrations of the MRN, ATM, ATM^P^, RPA and Rad51-Rad51par-BRCA2 complexes, respectively; *y*_12_ is the normalized level of incoming homologous sequence designated as $\left[{\mathrm{DNA}}_{\mathrm{inc}}\right]$; *y*_2_, *y*_5_, *y*_6_, *y*_8_, *y*_10_, *y*_11_, *y*_13_, *y*_14_ and *y*_15_ are the normalized intracellular concentrations of the [DSB•MRN-CtIP-ExoI-Dna2], $[\mathrm{DSB}•\mathrm{MRN}\text{-}\mathrm{CtIP}\text{-}\mathrm{ExoI}\text{-}\mathrm{Dna}2•{\mathrm{ATM}}^{P}]$, [ssDNA], [ssDNA•RPA], [ssDNA•RPA•Rad51-Rad51par-BRCA2], [Rad51 filament], $[\mathrm{Rad}51\;\mathrm{filament}•{\mathrm{DNA}}_{\mathrm{inc}}]$, [D-loop] and [dHJ] intermediate complexes, respectively; and *p_i_* are scaled rate constants. The variables are also normalized based on the Ku total cellular level as $y_{i}=Y_{i}/X_{1}$.

The dimensionless forms of the equations used in the SSA model are represented as follows:

(A6) $$\begin{array}{c} \frac{dz_{2}}{d\tau }=q_{1}y_{8}z_{1}-z_{2}(q_{-1}+q_{2}z_{2}^{2}),\\ \frac{dz_{3}}{d\tau }=q_{2}z_{{}_{2}}^{2}-q_{3}z_{3}z_{4}+q_{-3}z_{5},\\ \frac{dz_{5}}{d\tau }=q_{3}z_{3}z_{4}-z_{5}(q_{4}+q_{-3}),\\ \frac{dz_{6}}{d\tau }=q_{4}z_{5}-q_{5}z_{6}z_{7}+q_{-5}z_{8},\\ \frac{dz_{8}}{d\tau }=q_{5}z_{6}z_{7}-z_{8}(q_{6}+q_{-5}). \end{array}$$

The initial conditions of this system for wild-type cells are as follows: *z*_2_(0) = *z*_3_(0) = *z*_5_(0) = *z*_6_(0) = *z*_8_(0) = 0. The values of variables *z*_1_, *z*_4_ and *z*_7_ are set to be constant and equal to *x*_1_.

In Equation (A6), *z*_1_, *z*_4_ and *z*_7_ are scaled cellular levels of Rad52, ERCC1-XPF and LigIII enzymes, respectively; *z*_2_, *z*_3_, *z*_5_, *z*_6_ and *z*_8_ are scaled intracellular concentrations of the [ssDNA•RPA•Rad52], [Flap], [Flap•ERCC1-XPF], $\left[{\mathrm{dsDNA}}_{\mathrm{nicks}}\right]$ and $[{\mathrm{dsDNA}}_{\mathrm{nicks}}•\;\mathrm{LigIII}]\;$ intermediate complexes, respectively; and *q_i_* is the scaled rate constants. The variables are also normalized based on Ku total cellular level as $z_{i}=Z_{i}/X_{1}$.

The kinetics of the Alt-EJ intermediate complexes is described by the following system of ordinary differential equations:

(A7) $$\begin{array}{c} \frac{dw_{2}}{d\tau }=r_{1}w_{1}y_{6}-w_{2}(r_{2}+r_{-1}),\\ \frac{dw_{4}}{d\tau }=r_{2}w_{2}w_{3}-r_{3}w_{4},\\ \frac{dw_{5}}{d\tau }=r_{3}w_{4}-r_{4}w_{5}w_{6}+r_{-4}w_{7},\\ \frac{dw_{7}}{d\tau }=r_{4}w_{5}w_{6}-w_{7}(r_{5}+r_{-4}). \end{array}$$

The initial conditions of this system for wild-type cells are as follows: *w*_2_(0) = *w*_4_(0) = *w*_5_(0) = *w*_7_(0) = 0. The values of *w*_1_, *w*_3_ and *w*_6_ variables are set to be constant and equal to *x*_1_.

In Equation (A4), *w*_1_, *w*_3_ and *w*_6_ are scaled cellular levels of PARP1, Pol and LigI, respectively; *w*_2_, *w*_4_, *w*_5_ and *w*_7_ are scaled intracellular concentrations of the [ssDNA•PARP1] , [ssDNA•Pol] , [MicroHomol] and [MicroHomol•LigI] intermediate complexes, respectively; and *r_i_* is the scaled rate constants. The variables are also normalized based on Ku total cellular level as $w_{i}=W_{i}/X_{1}$.

## Appendix C. Parameters of the Model

The dimensionless parameters of Equation (A4) are $k_{1}=K_{1}X_{1}/K_{8}$, $k_{-1}=K_{-1}/K_{8}$, $k_{2}=K_{2}X_{1}/K_{8}$, $k_{-2}=K_{-2}/K_{8}$, $k_{3}=K_{3}/K_{8}$, $k_{4}=K_{4}X_{1}/K_{8}$, $k_{-4}=K_{-4}/K_{8}$, $k_{5}=K_{5}X_{1}/K_{8}$, $k_{-5}=K_{-5}/K_{8}$, $k_{6}=K_{6}X_{1}/K_{8}$, $k_{-6}=K_{-6}/K_{8}$, $k_{7}=K_{7}X_{1}/K_{8}$, $k_{-7}=K_{-7}/K_{8}$, $k_{8}=K_{8}/K_{8}=1$, $k_{9}=K_{9}/K_{8}$, $k_{10}=K_{10}/X_{1}$, $k_{11}=K_{11}/K_{8}$ and $k_{12}=K_{12}/K_{8}$. *K*_8_ is the rate of final NHEJ process resulting in the production of the repaired dsDNA and the unwinding of all repair factors. The reason for the selection of this constant as a scaling factor is the assumed independence of this parameter on possible variations due to the competition of repair pathways at earlier stages. This assumption is mainly important in terms of performing future modifications to the model, when rate constants of early stages are planned to connect with the damage complexity.

The scaled reaction rates in Equation (A5) are $p_{1}=P_{1}X_{1}/K_{8}$, $p_{-1}=P_{-1}/K_{8}$, $p_{2}=P_{2}/K_{8}$, $p_{3}=P_{3}X_{1}/K_{8}$, $p_{-3}=P_{-3}/K_{8}$, $p_{4}=P_{4}/K_{8}$, $p_{5}=P_{5}X_{1}/K_{8}$, $p_{-5}=P_{-5}/K_{8}$, $p_{6}=P_{6}X_{1}/K_{8}$, $p_{-6}=P_{-6}/K_{8}$, $p_{7}=P_{7}/K_{8}$, $p_{8}=P_{8}X_{1}/K_{8}$, $p_{-8}=P_{-8}/K_{8}$, $p_{9}=X_{1}/K_{8}$, $p_{10}=P_{10}/K_{8}$, $p_{11}=P_{11}/K_{8}$ and $p_{12}=P_{12}/K_{8}$. The scaling factors *X*_1_ and *K*_8_ are chosen to perform parameter normalization.

The scaled reaction rates in Equation (A6) are $q_{1}=Q_{1}X_{1}/K_{8}$, $q_{-1}=Q_{-1}/K_{8}$, $q_{2}=Q_{2}X_{1}/K_{8}$, $q_{3}=Q_{3}X_{1}/K_{8}$, $q_{-3}=Q_{-3}/K_{8}$, $q_{4}=Q_{4}/K_{8}$, $q_{5}=Q_{5}X_{1}/K_{8}$, $q_{-5}=Q_{-5}/K_{8}$ and $q_{6}=Q_{6}/K_{8}$. In this case, the same factors *X*_1_ and *K*_8_ are used for scaling rate constants.

The scaled reaction rates in Equation (A7) are $r_{1}=R_{1}X_{1}/K_{8}$, $r_{-1}=R_{-1}/K_{8}$, $r_{2}=R_{2}X_{1}/K_{8}$, $r_{3}=R_{3}/K_{8}$, and $r_{4}=R_{4}X_{1}/K_{8}$, $r_{-4}=R_{-4}/K_{8}$ and $r_{5}=R_{5}/K_{8}$. As in the other models, the same scaling factors *X*_1_ and *K*_8_ are chosen to perform parameter normalization.

The majority of the rate constants of enzymatic reactions were determined by fitting the corresponding model curves produced using Equations (A4)–(A7) to the experimental data regarding the kinetics of different stages of DSB repair. Other parameters characterizing the regularities of DSB induction were directly obtained from experimental measurements reviewed in the literature. For some of the parameters used, a dependence of their values on radiation dose is introduced. The functions representing these parameters are also shown in Table A1.

In order to estimate *α*(*L*), we used three sets of experimental data regarding DSB induction in GM38 and GM57 lines of human skin fibroblasts within the LET values ranging from 0.2 to 440 keV/µm [54,55,56]. The total DSB yield measured in these studies as Gy^−1^ per 10^9^ bp was recalculated to Gy^−1^ per cell, taking into account the fact that a diploid human cell in G_1_ phase contains about 5 × 10^9^ bp of DNA [54]. The experimental data were approximated using the exponential function α(L)=aexp(−bL) to obtain a continuous function within the stated LET range. The parameters of the function given in Table A1 were evaluated by adjusting the curve for *α*(*L*) to fit the results of the above-mentioned experimental measurements.

**Table A1:** Parameters of the model.

Parameter	Value	Parameter	Value
*a*	27.5	*P*_−5_	8.82 × 10^−5^ h^−1^
*b*	2.43 × 10^−3^	*P*_6_	1.87 × 10^5^ M^−1^ h^−1^
*K*_1_	11.05 M^−1^ h^−1^	*P*_−6_	1.55 × 10^−3^ h^−1^
*K*_−1_	6.6×10^−4^ h^−1^	*P*_7_	21.36 h^−1^
*K*_2_	$18.83\times (1.09-e^{-21.42/D^{1.82}})$ M^−1^ h^−1^	*P*_8_	1.20 × 10^4^ M^−1^ h^−1^
*K*_−2_	5.26 × 10^−1^ h^−1^	*P*_−8_	2.49 × 10^−4^ h^−1^
*K*_3_	1.86 M^−1^ h^−1^	*P*_9_	$1.11\times e^{6.16\times 10^{-6}/D^{2.68}}-D^{0.03}$ h^−1^
*K*_4_	$1.20+4.48\times 10^{5}\;e^{-12.70\;D^{0.09}}$ M^−1^ h^−1^	*P*_10_	7.20 × 10^−3^ h^−1^
*K*_−4_	3.86 × 10^−4^ h^−1^	*P*_11_	6.06 × 10^−4^ h^−1^
*K*_5_	15.24 M^−1^ h^−1^	*P*_12_	2.76 × 10^−1^ h^−1^
*K*_−5_	8.28 h^−1^	*Q*_1_	7.80 × 10^3^ M^−1^ h^−1^
*K*_6_	18.06 M^−1^ h^−1^	*Q*_−1_	1.71 × 10^−4^ h^−1^
*K*_−6_	1.33 h^−1^	*Q*_2_	3.00 × 10^4^ M^−1^ h^−1^
*K*_7_	2.73 × 10^5^ M^−1^ h^−1^	*Q*_3_	6.00 × 10^3^ M^−1^ h^−1^
*K*_−7_	3.20 h^−1^	*Q*_−3_	6.06 × 10^−4^ h^−1^
*K*_8_	5.52 × 10^−1^ h^−1^	*Q*_4_	1.66 × 10^−6^ h^−1^
*K*_9_	1.66 × 10^−1^ h^−1^	*Q*_5_	8.40 × 10^4^ M^−1^ h^−1^
*K*_10_	1.93 × 10^−7^/*N_ir_* M	*Q*_−5_	4.75 × 10^−4^ h^−1^
*K*_11_	7.50 × 10^−2^ h^−1^	*Q*_6_	11.58 h^−1^
*K*_12_	11.10 h^−1^	*R*_1_	2.39 × 10^3^ M^−1^ h^−1^
*P*_1_	1.75 × 10^3^ M^−1^ h^−1^	*R*_−1_	12.63 h^−1^
*P*_−1_	1.33 × 10^−4^ h^−1^	*R*_2_	4.07 × 10^4^ M^−1^ h^−1^
*P*_2_	7.21 h^−1^	*R*_3_	9.82 h^−1^
*P*_3_	1.37 × 10^4^ M^−1^ h^−1^	*R*_4_	1.47 × 10^5^ M^−1^ h^−1^
*P*_−3_	2.34 h^−1^	*R*_4_	12.30 h^−1^
*P*_4_	5.52 × 10^−2^ h^−1^	*R*_−4_	2.72 h^−1^
*P*_5_	1.20 × 10^5^ M^−1^ h^−1^	*R*_5_	1.65 × 10^−1^ h^−1^
*N*_irrep_	$\left\{\begin{array}{l} 0.12\;e^{-2.48\;D^{2.02}}-0.11\;e^{-5.43\;D^{0.76}},\;D<1\\ 0.01,\;D\geq 1 \end{array}\right.$		
